# Genome Sequence of *Oenococcus oeni* OM27, the First Fully Assembled Genome of a Strain Isolated from an Italian Wine

**DOI:** 10.1128/genomeA.00658-14

**Published:** 2014-07-03

**Authors:** Antonella Lamontanara, Luigi Orrù, Luigi Cattivelli, Pasquale Russo, Giuseppe Spano, Vittorio Capozzi

**Affiliations:** a Consiglio per la Ricerca e Sperimentazione in Agricoltura (CRA), Genomic Research Centre, Fiorenzuola d’Arda, Italy; b Department of Agriculture, Food and Environment Sciences, University of Foggia, Foggia, Italy

## Abstract

*Oenococcus oeni* OM27 is a strain selected from “Nero di Troia” wine undergoing spontaneous malolactic fermentation. “Nero di Troia” is a wine made from “Uva di Troia” grapes, an autochthonous black grape variety from the Apulian region (south of Italy). In this paper we present a 1.78-Mb assembly of the *O. oeni* OM27 genome, the first fully assembled genome of an *O. oeni* strain from an Italian wine.

## GENOME ANNOUNCEMENT

*Oenococcus oeni* OM17 is a strain isolated from “Nero di Troia” wine undergoing spontaneous malolactic fermentation (MLF) (1). “Nero di Troia” is a typical Apulian (Italy) red wine obtained from “Uva di Troia,” an autochthonous black grape variety. *O. oeni* is the main lactic acid bacterium species involved in the microbial decarboxylation of L-malic acid into L-lactic acid and CO_2_ that usually takes place in wine after the alcoholic fermentation of grape must. This bio-conversion improves the organoleptic properties of wine (2–4). The only *O. oeni* complete genome sequence available is for *O. oeni* PSU-1 (5), while the assembled genome sequences of 13 *O. oeni* strains were recently deposited in the GenBank database (6). For *O. oeni* strain ATCC BAA-1163, a proteome reference map is also available (7). Recently, the role of cultivar, vintage, and climate in the microbial biogeography of wine grapes has been clarified, corroborating the existence of a “microbial terroir” for wine grapes (8, 9), an important concept for fermented food and beverages with a geographical indication status (10–13). *O. oeni* OM27 is a good candidate to design autochthonous starter cultures to perform MLF in wine obtained from “Uva di Troia” grapes. The assembled complete genome represents an important tool to investigate the strain safety (14–16), the tolerance to the hostile condition of wine (17, 18), and the molecular basis of the positive contribution to the final product (3, 4, 13, 19). To the best of our knowledge, *O. oeni* OM27 is the first published fully assembled genome of an *O. oeni* strain from an Italian wine.

2 µg of genomic DNA was subjected to library preparation using the TruSeq DNA sample prep kit FC-121-1001 according to the manufacturer’s instructions. Whole-genome sequencing of *O. oeni* OM27 was performed using the Illumina GAIIx platform. Prior to assembly, raw reads were filtered using PRINSEQ v0.20.3 software (20). This filtering step was performed in order to remove the 3′ ends showing a quality score below 25 (*Q*<25), the reads containing a percentage of uncalled based (“N” characters) equal or greater than 10% and the duplicated sequences. After filtering, a total of 20,403,000 paired end reads of 115-bp length were obtained corresponding to a coverage of about 1,300× (the *O. oeni* OM27 size is about 1.78 Mb). Genome sequences were *de novo* assembled using the Ray v2.2.0 assembly program (21) with default parameters and using a k-mer size of 71. The assembly resulted in 20 contigs with an *N*_50_ length of 184,677 bp. The size of the shortest contig was 1,255 bp while the length of the longest contig was 550,977 bp. The sequence was annotated by the National Center for Biotechnology Information (NCBI) Prokaryotic Genomes Annotation Pipeline.

The genome is 1,786,146 bp long with a G+C content of 37.9%. The genome size and the G+C content are comparable to those of the other published *O. oeni* strains (http://www.ncbi.nlm.nih.gov/genome/genomes/541). Out of 1,810 predicted genes, 1,682 were protein coding genes, 80 were annotated as pseudo-genes, while 48 were RNA coding genes (42 tRNAs and 6 rRNAs).

### Nucleotide sequence accession number.

The whole-genome shotgun project of *O. oeni* OM27 has been deposited at DDBJ/EMBL/GenBank under accession no. JMIS00000000.
